# Psychometric Properties of the Turkish Version of the University of Virginia Parent Low Blood Sugar Survey

**DOI:** 10.4274/jcrpe.5028

**Published:** 2018-05-18

**Authors:** Nesrin Şen Celasin, Çağrı Çövener Özçelik, Şükriye Şahin

**Affiliations:** 1 Manisa Celal Bayar University Faculty of Health Sciences, Division of Nursing, Department of Pediatric Nursing, Manisa, Turkey; 2 Marmara University Faculty of Health Sciences, Division of Nursing, Department of Pediatric Nursing, İstanbul, Turkey; 3 Marmara University Faculty of Health Sciences, Division of Nursing, Department of Fundemantals of Nursing, İstanbul, Turkey

**Keywords:** Type 1 diabetes mellitus, adolescent, validity, reliability, Turkey

## Abstract

**Objective:**

The aim of this study was to produce and validate a Turkish version of the University of Virginia Parent Low Blood Sugar Survey (P-LBSS). The P-LBSS is used to assess parental fear of their diabetic children’s hypoglycemia.

**Methods:**

Linguistic, content and face validity of the translated P-LBSS was tested. Afterwards, explanatory and confirmatory factor analyses were conducted in order to evaluate construct validity.

**Results:**

The sample included 390 parents of type 1 diabetic adolescents aged 12-17 years. Results of the factor analysis showed that the Turkish P-LBSS had 2 subscales (behavior and worry) as in the original. The Cronbach’s alpha coefficient of the Turkish version of the total P-LBSS was found to be 0.803, and the value was 0.865 for the behavior and 0.790 for the worry subscales. Psychometric investigation of the Turkish version of P-LBSS indicated high reliability and good retestability, content and construct validity.

**Conclusion:**

The Turkish P-LBSS is a valid and reliable instrument to measure the fear of hypoglycemia experienced by parents of diabetic adolescents aged between 12-17 years in the Turkish population.

## What is already known on this topic?

It is well known that fear of hypoglycemia causes various problems in achieving metabolic control.

## What this study adds?

This study showed that the Turkish Parent Low Blood Sugar Survey would aid pediatric diabetes nurses in Turkey in the evaluation of the fear of hypoglycemia experienced by parents.

## Introduction

Type 1 diabetes mellitus (T1DM) mostly presents during childhood and adolescence (1,2). Although seen in every age group, T1DM usually presents between 7 and 15 years of age (3). Factors such as having a chronic disease, poor adaptation to disease, lifestyle changes, the burden of disease on social life, lowered self-esteem, and fear of complications may negatively affect diabetes management (4,5).

Despite the recent progress in the treatment of T1DM, children and adolescents diagnosed with it are at risk of psychological problems due to the difficulties of diabetes management, such as diet adherence, blood glucose monitoring, insulin administration, physical exercise and self-care (5). Having diabetes brings about changes for the child and his/her family in their daily activities and lifestyle (5,6,7,8,9,10,11,12).            

Hypoglycemia is the most commonly seen acute complication in children with T1DM and partially results from intense treatment regimes (13,14,15,16,17). Although transient, hypoglycemia, if untreated, may result in serious morbidity (16,18,19,20). Fear of hypoglycemia negatively affects quality of life (15,21,22,23). Patients and families have been reported to deliberately keep blood glucose levels high, despite having been informed of the risks, using strategies such as decreasing insulin doses, overeating, limiting activities and measuring blood glucose very often (16,19,22,24,25,26). It has been reported that parents may measure blood glucose repeatedly at night, increasing both patient and parental stress (15,17,27,28,29).

Diabetes training nurses have a major role to play in educating families and alleviating the fear of hypoglycemia and provide support to affected families (1,30,31). The Virginia Parent Low Blood Sugar Survey (P-LBSS) was developed by Gonder-Frederick et al (16) to assess parental attitudes to childrens’ hypoglycemia.

The aim of this study was to assess a Turkish adaptation of the P-LBSS and to evaluate the fear of hypoglycemia experienced by parents of a large cohort of T1DM adolescents.

## Methods

Methodological design was used to determine the validity and reliability of instruments in order to measure constructs used as variables in research (32).

**Virginia P-LBSS:** The P-LBSS consists of 25 questions in two subscales: the behavior subscale (questions 1-10) and the worry subscale (questions 11-25). Responses are scored on a Likert type scale (never: 0, rarely: 1, sometimes: 2, often: 3, almost always: 4). Total scores range between 0-100 with higher total scores indicating increased fear of hypoglycemia (16). The Cronbach’s alpha reliability coefficients of the original total scale, the behavior subscale, and the worry subscale were found to be 0.89, 0.76 and 0.91, respectively, indicating good reliability. 

The questionnaire was translated into Turkish by two competent English teachers. In order to test content validity, the scale was assessed for comprehensibility by 18 experts. The experts scored each item on a scale of 1 to 4 in accordance with the Davis technique (1992) (33). Finally, the scale was completed by 15 adolescents to evaluate scale legibility. Small corrections were made to achieve face validity. In addition, a Parent Identification Form was prepared in line with the literature (1,14,30) which consisted of 15 items with seven open ended and eight closed questions about sociodemographic characteristics.

Parents of adolescents aged between 12-17 years with T1DM were invited to participate in this study. Subjects were recruited from five hospitals, three in İstanbul and two in İzmir. Since adaptation to a chronic disease takes up to one year, parents of adolescents with a diabetes duration of one year or more, who regularly received routine three monthly follow-up care, were invited to participate. Sample size for questionnaire adaption studies should be between twice and ten times the number of questions included (34,35,36). The P-LBSS used in this study has 25 items and the aim was to recruit at least 250 families. 

The data were collected between March and June 2016. Completion of the test took 15 to 20 minutes. For test retest reliability, the scale was repeated by the same parents three weeks later.

### Ethical Considerations

Ethical board permission was taken from the Marmara University Health Sciences Institute Ethical Board (IRB no: 26.10.2015-14). Written permission from the institutions where the study would be conducted were obtained. Prior to data collection, the participants were informed about the study. Parents who agreed to participate in the study gave written informed consent.

### Statistical Analysis

Number Cruncher Statistical System 2007 (Kaysville, Utah, USA) was used for statistical analyses. The expert views on the content validity of the scale were evaluated using the content validity index (CVI) (36). In order to evaluate structure validity, confirmatory and explanatory factor analyses were performed. In the reliability analysis, the Cronbach’s alpha values and item-total score correlation coefficients were calculated. In test-retest reliability, the Intraclass Correlation Coefficient (ICC) was calculated. Descriptive statistical analyses [mean, standard deviation (SD), percentages] were also used. Data was evaluated at a 95% confidence interval. Significance level was set at p<0.05.

## Results

Mean age of the mothers and fathers was 42.44±6.44 (range 29-56) and 46.37±6.51 (28-56) years respectively. Mean ± SD time since diagnosis was 5.96±3.47 (range 1-17) years. Mean hemoglobin A1c value was found to be 8.96±1.82%. Expert assessment of the questionnaire by CVI scoring gave a maximum value of 1.00. 

**Construct validity:** Explanatory and confirmatory factor analyses were performed to test construct validity.

**Explanatory factor analysis: **The Kaiser-Meyer-Olkin (KMO) coefficient was found to be 0.881. Thus, it was shown that the sample size was sufficient for factor analysis. The Bartlett’s sphericity test was found to be significant (p<0.05), showing that the data set had multivariate normality (37,38). The result of the Bartlett’s sphericity test in this study was found to be χ^2^=3630, df=325,p=0.001. A Varimax rotation was used and the scale was found to have two factors as in the original scale, factor 1 being the behavior subscale and factor 2 the worry subscale. Factor loadings in factor 1 were found to range between 0.315 and 0.879 and in factor 2 between 0.304 and 0.638 (Table 1). The two-factor structure explained 39.1% of the total variance. Factor loadings were acceptable (Table 1).

**Figure 1:**
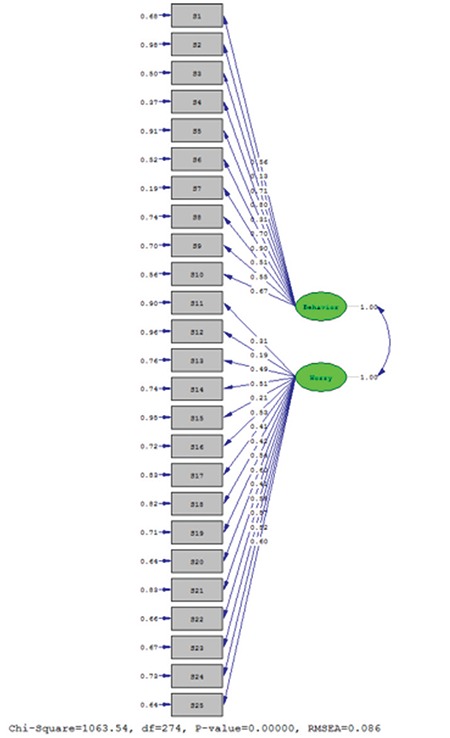
Path diagram of confirmatory factor analysis for University of Virginia Parent Low Blood Sugar Survey

**Confirmatory factor analysis:** Confirmatory factor analysis confirms the factors determined in explanatory factor analysis (39). For the structural validity of a scale to be confirmed, the “Goodness of fit statistics”, which can be obtained via confirmatory factor analysis, should be at acceptable levels. In the current study, the factor model fit the data (p<0.001) and the Root Mean Square Error of Approximation (RMSEA) was 0.086 (Figure 1). The χ^2^/df index was found to be 3.88.

**Reliability: **Item-total correlations were found to vary between 0.019 and 0.595 (Table 1). The Cronbach’s alpha coefficients of the scale are given in Table 2. The test-retest reliability results are given in Table 3. According to the results of the ICC analysis, the level of consistency between the first and last measurements was 92.4% for the behavior subscale, 74.9% for the worry subscale and 82.5% for the total scale.

## Discussion

The main characteristics sought in a good instrument are reliability and validity (32). In the current study, the psychometric properties of the Turkish version of the P-LBSS were examined in detail. 

Content validity represents the universe of content or the domain of given constructs (40,41). A CVI value above 0.80 indicates good content validity (36). In the present study, the CVI was found to be 1.00 indicating excellent content validity.

In the current study, the KMO and the Bartlett’s sphericity test were used to evaluate sample size adequacy. KMO may range from 0 to 1, with higher values indicating appropriate sample size (34,38). In the literature, KMO values between 0.80 and 0.89 reflect a “very good” sample size (35).

In this study, the KMO was found to be 0.881, indicating sample size adequacy. The Bartlett’s sphericity test was used to test the hypothesis that correlation matrices were similar, and this hypothesis was rejected at a value of p=0.001. The results of the Bartlett’s test being p<0.01 showed that measurement was not affected by the sample size and that the sample size was adequate for factor analysis.

Construct validity was tested using explanatory and confirmatory factor analysis. The factor analytical approach is a procedure that provides information about the extent to which a set of items measure the same underlying construct (40,41). 

The Varimax rotation method showed that the two-factor structure explained 39.1% of the total variation. Higher variance rates indicate a stronger factor structure.

Factor loadings of items in factor 1 ranged between 0.315 and 0.879, and in factor 2 between 0.304 and 0.638. Factor loadings should be a minimum of 0.30 in scale development and adaptation to discriminate between the characteristics to be measured (38), thus adequate discrimination was present. In the interpretation of goodness of fit indices in confirmatory factor analysis, the RMSEA fit index is used to assess goodness of fit indices in confirmatory factor testing with 0<RMSEA<0.05 showing good fit, while 0.05≤RMSEA≤0.10 shows acceptable fit (38,42). The RMSEA value of our instrument was 0.086 indicating acceptable fit. Similarly, a χ^2^/df fit index of ≤3 shows perfect fit and ≤5 shows good fit (43). The χ^2^/df value for our questionnaire was 3.88 indicating good fit.

Reliability of an instrument refers to the extent to which the instrument yields consistent results on repeated measures (40). There are five major techniques for reliability testing. These techniques include test-retest reliability, parallel or alternate forms, item-total correlation, split-half reliability, Kuder-Richardson-20, Cronbach’s alpha, and inter-rater reliability (40). In the current study, the Cronbach’s alpha coefficient, item-total correlations and test-retest reliability were used. In scale development and adaptation, scales with Cronbach’s alpha values at and above 0.70 are accepted as reliable (38). Accordingly, the Cronbach’s alpha reliability coefficients of the total scale and the behavior and worry subscales were found to be acceptable (Table 2). These values showed that the scale is a reliable instrument and parallel results were obtained with the original scale.

Item-total correlations show the reliability of each item in a scale (36). An item total score correlation of 0.30 and above shows that the items are adequate for measuring the desired characteristic and that the items are consistent with the total scale (38,39). In the present study, the item-total correlations ranged from 0.019 to 0.595 with four questions giving values below 0.30. However, since the factor structure was tested using confirmatory factor analysis, and since the Cronbach’s alpha coefficient of the scale was above 0.70, the original structure of the scale was maintained. Thus, the four items were not removed from the scale.

Test-retest reliability is used for evaluating the consistency of the scale over time, with values above 0.70 indicating good retest reliability (38). The correlation values obtained in this study indicated perfect correlation and demonstrated the consistency of scale scores over time (Table 3).

The usability of the scale should also be tested in parents of adolescents with a diabetes duration of less than one year and adolescents with co-morbid disorders such as celiac disease or hypothyroidism.

## Conclusion

The Turkish version of the P-LBSS had high reliability and good content and construct validity. The Turkish P-LBSS is a valid and reliable instrument to measure the fear of hypoglycemia experienced by parents of T1DM adolescents in the Turkish population. Additionally, the P-LBSS, which is easy for pediatric diabetes nurses to use, can help in evaluating parental fear of childrens’ hypoglycemia. Thus, appropriate psychological help could be provided. Use of this questionnaire may have the effect of improving the quality of adolescent diabetic nursing care in Turkey.

## Figures and Tables

**Table 1:**
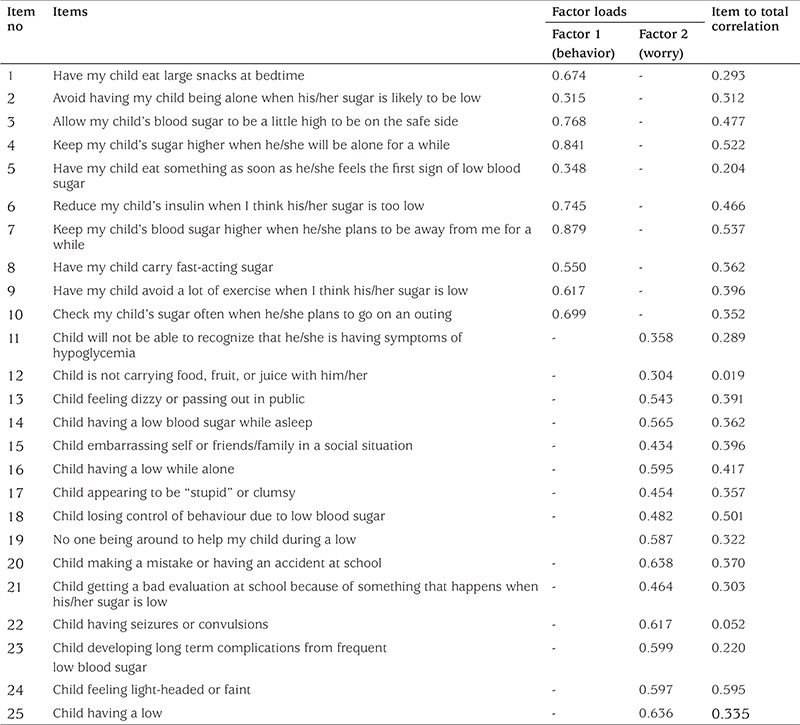
Factor Loads and Item to total correlation of University of Virginia Parent Low Blood Sugar Survey

**Table 2:**
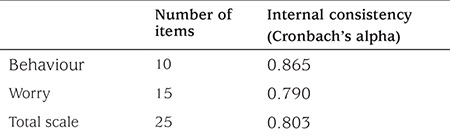
Internal consistency of University of Virginia Parent Low Blood Sugar Survey

**Table 3:**
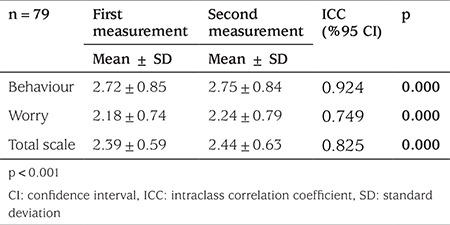
Test-retest reliability of University of Virginia Parent Low Blood Sugar Survey
